# Constraints and spandrels of interareal connectomes

**DOI:** 10.1038/ncomms13812

**Published:** 2016-12-07

**Authors:** Mikail Rubinov

**Affiliations:** 1 Behavioural and Clinical Neuroscience Institute, Department of Psychiatry, University of Cambridge, Cambridge CB2 3EB, UK; 2 Janelia Research Campus, Howard Hughes Medical Institute, Ashburn, Virginia 20147, USA

## Abstract

Interareal connectomes are whole-brain wiring diagrams of white-matter pathways. Recent studies have identified modules, hubs, module hierarchies and rich clubs as structural hallmarks of these wiring diagrams. An influential current theory postulates that connectome modules are adequately explained by evolutionary pressures for wiring economy, but that the other hallmarks are not explained by such pressures and are therefore less trivial. Here, we use constraint network models to test these postulates in current gold-standard vertebrate and invertebrate interareal-connectome reconstructions. We show that empirical wiring-cost constraints inadequately explain connectome module organization, and that simultaneous module and hub constraints induce the structural byproducts of hierarchies and rich clubs. These byproducts, known as spandrels in evolutionary biology, include the structural substrate of the default-mode network. Our results imply that currently standard connectome characterizations are based on circular analyses or double dipping, and we emphasize an integrative approach to future connectome analyses for avoiding such pitfalls.

## Abstract (web summary)

Whole-brain networks of long-range neuronal pathways are characterized by interdependencies between structural features. Here the author shows that module hierarchy and rich club features in these networks are structural byproducts (spandrels) of module and hub constraints, but not of wiring-cost constraints.

The connectome is the complete structural wiring diagram of the brain or nervous system and an important substrate of brain function^1^. Understanding the principles of connectome organization is a grand challenge in systems neuroscience. Such understanding requires the simultaneous reconstruction of accurate whole-brain wiring diagrams, the reduction of these diagrams to informative features, such as anatomical maps of sensory, association and motor systems, and the description of biologically valid mechanisms underlying the organization of these features.

Interareal connectomes^2^,^3^ are wiring diagrams of whole-brain interareal white-matter pathways, and are the only currently reconstructible connectomes of vertebrate and invertebrate organisms. (As an aside, we note that such diagrams have also been termed mesoscale connectomes^4^ or macroscale connectomes^5^; we use the term interareal connectomes to sidestep this terminological ambiguity. These diagrams have also been termed projectomes, to emphasize the relatively coarse and non-synaptic nature of the observed connectivity^6^; but the adoption of this term remains uncertain^7^.)

Two recent reconstructions of interareal connectomes, based on data generated by the Allen Institute mouse brain initiative^4^,^8^ and the FlyCircuit *Drosophila* brain initiative^9^,^10^, have an unmatched combination of brain-wide coverage (compared with more spatially limited imaging studies^3^,^11^,^12^), single-site acquisition (compared with meta-analyses of heterogeneous studies^5^,^13^) and high-resolution light-microscopy imaging (compared with lower quality diffusion MRI^14^,^15^). By virtue of this combination, these data sets represent the current gold-standard interareal connectome reconstructions of model vertebrate and invertebrate organisms.

Network science is an intuitive framework for analysis of connectome reconstructions^16^. This framework represents connectomes as mathematical graphs (networks of nodes and connections) and uses graph algorithms to detect interesting connectome features^17^. Early network analyses of interareal connectomes described modules and hubs as two putative hallmarks of connectome organization. Modules are densely intra-connected groups of areas, thought to form functionally specialized systems^18^, while hubs are strongly or diversely connected areas, thought to integrate information between such systems^19^. Studies reported the simultaneous presence of modules and hubs as a ‘small-world' network organization^20^.

Recent analyses identified module hierarchies and rich clubs as two additional putative connectome hallmarks. Module hierarchies are nestings of smaller modules within larger modules, thought to facilitate diverse functional brain states^21^. Module hierarchies are distinct from sensory-motor hierarchies, although they likewise represent arrangements of progressively more specialized neural functional units^22^. In contrast, rich clubs are densely intra-connected groups of hub areas, thought to reflect the dense connectivity between association areas and represent a backbone of functional brain integration^23^,^24^. One prominent rich club is the structural substrate of the default-mode network^25^, a system implicated in human cognition (including self-referential processing, theory of mind and autobiographical memory^26^), and brain disorders (including depression, schizophrenia and autism^27^), but also present in mice^28^ and rats^29^. Figure 1 and Supplementary Table 1 illustrate and summarize module hierarchies and rich clubs described in recent analyses of the mouse and fly interareal connectome reconstructions^8^,^10^.

An influential current theory, inspired by Ramón y Cajal's principles for conservation for time, space and material^30^, postulates that the above connectome hallmarks arise from competing selection pressures for economical functional segregation and efficient functional integration^31^. Specifically, this theory postulates that wiring-economy pressures explain the presence of segregative modules, but not the presence of integrative hubs, module hierarchies and rich clubs. Studies in support of this theory show that wiring cost, a marker of white-matter tract length and capacity, is low but not minimized in interareal connectomes, that minimized-wiring-cost models of connectomes are modular but not integrative; and that generative models which reconcile wiring cost and functional integration accurately predict global and specific features of connectome organization^8^,^32^,^33^,^34^,^35^,^36^,^37^,^38^,^39^,^40^,^41^.

However, three important aspects of such studies are not empirically grounded and thus difficult to interpret. First, minimized-wiring-cost spatial lattices have unrealistically low wiring costs, and are therefore artificial connectome representations. Second, generative models based on phenomenological rules—such as preferential assignment of connections between similar regions, or optimization of trade-offs between wiring-cost and efficiency—are hard to validate in interareal connectomes. Third, global connectome features—such as the modularity (an anatomically nonspecific marker for the existence of modules), node-centrality distributions (ditto hubs), small-world-ness (ditto modules and hubs) and network motifs (ditto patterns of recurring connectivity)—describe that the connectome is generically complex, but do not specify where this complexity is instantiated.

Here, we adopt a qualitatively different, empirically grounded, approach to the study of connectome organization. We assume the existence of basic hallmarks, a set of anatomically specific connectome features selected for independent functionality, and study the effect of structural constraints induced by the simultaneous presence of these features^42^,^43^. We hypothesize that structural constraints are bound to induce a host of structural byproducts^44^,^45^. Gould and Lewontin famously compared such byproducts with spandrels, triangular spaces induced by constraints of arches and rectangular frames^46^,^47^, and this metaphor directly describes the effect we seek to investigate (Fig. 2). We contrast this approach with reductionist analyses which assume the independent functionality of all observed features^46^, and which consequently risk engaging in circular analyses or double dipping^48^.

We study constraint network models, networks which satisfy empirical constraints but are otherwise maximally random. In other words, we do not seek to phenomenologically generate connectome-like networks, but rather to sample uniformly or unbiasedly—and therefore independently of generative mechanisms—the space of all networks with *a priori* specified constraints, and thus to study the effect of these constraints on other features of connectome organization. The robust presence of higher-order (not explicitly constrained) features in such networks implies that these higher-order features are byproducts induced by structural constraints (that is, spandrels). Current connectome studies typically constrain only node properties, and mostly in simplified binary, undirected and sparse connectome models^49^. Here, we immensely improve the utility and applicability of constraint network models by adapting methods for constraining a wide and potentially arbitrary range of observed features in weighted, directed and dense connectome reconstructions. This improvement allows us to directly test and strongly challenge the major current notions of interareal connectome organization, underscoring the importance of this type of analysis for future connectome studies.

## Results

### Overview of the general approach

The key steps in our approach are the choice of basic connectome hallmarks, the uniform sampling of networks constrained by these hallmarks, and the evaluation of sampled network accuracy. We now briefly describe each of these steps in turn.

Our first step is the choice of basic connectome hallmarks. This choice is equivalent in spirit to the formulation of postulates or axioms essential for construction of systems of knowledge in any scientific field, including in biology^50^. Previous studies considered exponential-family or power-law relationships between physical distance and connection weight to be the candidate basic connectome hallmarks^8^,^40^,^51^,^52^. Below we examined similar relationships in nonparametric wiring-cost models, but found that such relationships did not determine important aspects of connectome organization.

In contrast, we assumed that modules and hubs, the canonical forms of segregation and integration, represent such basic hallmarks of interareal connectomes. We denoted the anatomical composition of modules and hubs through intra-module weight and node-strength constraints (see ‘Methods' section for mathematical definitions). We inferred these features from the data and thus ensured that our assumptions are empirically grounded. At the same time, and in contrast to more data-driven approaches^53^, these assumptions are biological hypotheses, testable and falsifiable in future analyses, as we discuss below. In principle, modules and hubs may underpin the organization of other complex systems, but such assumptions need to be made on a domain-specific basis.

Our second step is the uniform sampling of networks with the specified empirical constraints. Uniform sampling is an important part of our approach, as it focuses the investigation on the basic hallmarks, and away from phenomenological generative mechanisms. However, it is hard to uniformly sample networks with all but the simplest constraints (it is unlikely that such sampling can be achieved in polynomial time). Here, we used two methods, with complementary strengths and weaknesses, to approximate such sampling with distinct strategies (see Table 1 and ‘Methods' section for more details).

Our primary method^54^ is based on minimization of constraint errors with simulated annealing, a metaheuristic numerical optimization algorithm. This method samples networks with hard constraints, such that each network satisfies the constraints with high accuracy; we ensured that constraint errors were low (normalized constraint error <0.005) and similar for all sampled network models (Supplementary Fig. 1). We focused on results obtained with this method, due to its potential to uniformly sample networks with arbitrary constraints. However, it is important to note that despite this potential, such uniformity of sampling is not formally guaranteed.

Our alternative sampling method^55^ is based on maximum-likelihood estimation, an exact procedure for computing network probabilities. This method unbiasedly samples networks with soft constraints such that the constraints are satisfied on average in the full network ensemble, but not, in general, in each individually sampled network^56^,^57^. However, it is important to note that sampling with soft constraints may not accurately approximate the target distributions we wish to sample, for instance if such distributions are multimodal.

Our third step is the evaluation of accuracy with which the sampled networks reproduced the empirical module hierarchies and rich clubs (Supplementary Table 1). We evaluated the accuracy of module hierarchies with the normalized mutual information (NMI) between high- and low-resolution module and connectome partitions. This measure of partition similarity ranges from 1 for identical partitions to 0 for maximally different partitions, or in the absence of stable partitions. We accounted for inaccuracies due to variability of individual samples by comparing our results with a hierarchical benchmark model, a model with constrained high- and low-resolution intra- and inter-module weight, as well as node strength (Supplementary Fig. 2c). In practice, this benchmark provided an upper bound on the accuracy of hierarchical structure in our studied models.

We evaluated the existence of empirical rich clubs by computing the model/connectome empirical rich-club density for each model. A model lacks a rich club when this density is much lower than one, and possesses a rich club otherwise. The rich-club density, unlike the NMI, is a physical quantity, such that variability of individual samples averages out in the network ensemble; in practice this makes comparison with a benchmark model less necessary.

We reported all results as medians (interquartile ranges) estimated from 100 network samples for each type of model[Fig f3].

### Wiring cost and module organization

Current notions consider low wiring cost to represent an accurate proxy of module organization. Indeed, minimized wiring-cost spatial lattices, network models constructed by assigning progressively stronger weights to shorter pathways, had a relatively high NMI with high-resolution connectome module partitions (NMI: mouse 0.68 (0.68–0.69), fly: 0.62 (0.60–0.62); Fig. 4a,b). Nonetheless the module organization of these lattices was less accurate than that of the benchmark models (NMI mouse 0.84 (0.81, 0.87), fly: 0.84 (0.79, 0.86); Supplementary Fig. 3a,b). Qualitatively, the accuracy was highest for the more peripheral and well-delineated modules, such as the brainstem and cerebellar modules of the mouse, and the visual module (optic lobe) of the fly, and lowest for the more central and spatially intertwined modules, such as the olfactory and hippocampal modules of the mouse, and the premotor module (central complex) of the fly.

Spatial lattices are artificial models with unrealistically low wiring cost, and it is generally assumed that modifications of these lattices which increase wiring cost to realistic levels (for example, through inclusion of long and costly inter-module connections), have little effect on module organization. However, this assumption is typically tested with phenomenological models. Here, we found that this assumption is inaccurate; network models with empirical levels of wiring cost had consistently low intra-module weight (Fig. 3b,c), and a low NMI with high-resolution module partitions in both connectomes (NMI mouse: 0.43 (0–0.50), fly: 0.48 (0.39–0.53); Fig. 4a,b). These results suggest that realistic wiring-cost constraints, with no additional assumptions, represented an insufficient determinant of module organization.

### Basic hallmarks and structural byproducts

In contrast, we adopted the intra-module weight of high-resolution modules as a more direct constraint of module organization. We introduced the basic-hallmark model, a network model with constrained intra-module weight and node strength (Fig. 3d and ‘Methods' section). The basic-hallmark model, unlike wiring-cost constrained models, reproduced the high-resolution module organization of both connectomes (NMI mouse: 0.85 (0.84–0.87), fly: 0.84 (0.79–0.88); Fig. 4a,b) with accuracy comparable to the benchmark model (above), and thus allowed us to study more directly the effects of combined module and hub constraints on other connectome features.

The basic-hallmark model reproduced much of the (not explicitly constrained) low-resolution module organization of both connectomes (NMI mouse: 0.71 (0.68–0.75), fly: 0.71 (0.64–0.76); Fig. 4a,b) with accuracy comparable to, or lower than, the benchmark model (NMI mouse: 0.75 (0.71, 0.81), fly 0.89 (0.83, 0.92); Supplementary Fig. 3a,b). These results suggest that module and hub constraints induced module hierarchies, likely through a better approximation of inter-module weights (Supplementary Fig. 2d). This effect is nontrivial, and low-resolution modules were less well reproduced with other models, such as models with constrained wiring cost and node strength (NMI mouse: 0.22 (0–0.35), fly: 0.18 (0.17–0.30); Fig. 4a,b), and a model with constrained intra-module weight but not node strength (NMI mouse: 0.30 (0.30–0.46), fly: 0.50 (0.46–0.56); Supplementary Fig. 3a,b). In general, we noted smaller effect sizes for the fly connectome, consistent with its less well-resolved connectome reconstruction (44% of nodes and 30% of connections of the mouse connectome reconstruction); we posit that such effect sizes will increase in more highly resolved reconstructions.

**Figure 1:**
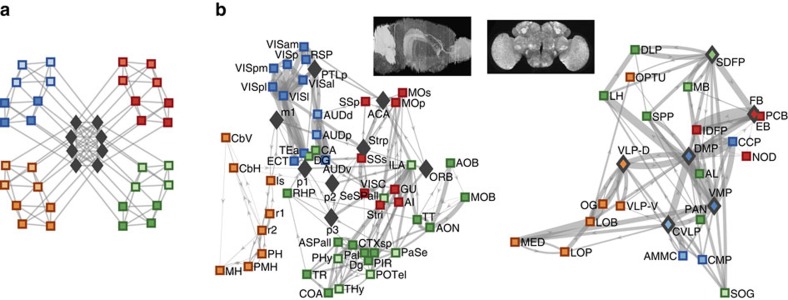
Convergent evidence for module hierarchies and rich clubs in mouse and fly interareal connectome reconstructions. (**a**) An idealized network representation of module hierarchies and rich clubs. Module hierarchies are nestings of high-resolution modules in low-resolution modules: here, each of the four low-resolution modules (blue-, green-, red- and brown-node borders) contains two four-node high-resolution modules (dark- and light-node centres). Rich clubs are densely intra-connected groups of hub areas: here, a group of central areas (black diamonds) equally connect to all modules and strongly interconnect between themselves. (**b**) Graph representations of mouse (left) and fly (right) connectome reconstructions. Nodes of one hemisphere for each connectome are drawn at anisotropically scaled areal centres of mass (cf. inset isotropically scaled views of brain maximal projections). The two strongest in- and out- projections for each node are shown as lines (projection weights are shown as line widths). Both connectomes had a two-level module hierarchical organization, with seven high-resolution modules nested inside four low-resolution modules. In the mouse connectome, the low-resolution modules comprise the brainstem/cerebellar (brown), visual/auditory (blue), somatosensory/somatomotor (red) and olfactory/hippocampal (green) modules. In the fly connectome, the low-resolution modules comprise the visual (brown), auditory/mechanosensory (blue), pre-motor (red) and olfactory (green) modules. Both connectomes had two rich clubs. In the mouse connectome, the auditory-visual rich club contained nodes with high connection strength, largely located in the auditory-visual module, while the default-mode rich club (diamonds) contained nodes with high connection diversity which could not be assigned to specific modules; these nodes represent the mouse structural substrate of the default-mode network. In the fly connectome, rich clubs were reported as nested shells: an inner shell of high-strength areas spanning auditory/mechanosensory and visual modules was nested in an outer shell (diamonds) which additionally included nodes from olfactory and premotor modules. See Supplementary Table 1 for areal abbreviations and complete definitions of module hierarchies and rich clubs for both connectome reconstructions.

The basic-hallmark model additionally, and in contrast to simpler models, reproduced all rich-club densities in both connectomes (Fig. 4c,d and Supplementary Fig. 3c,d), namely the visual-auditory rich club (model/connectome rich-club density: 0.95 (0.92–0.97)) and the default-mode rich club (density: 1.04 (0.96–1.12)) of the mouse connectome, as well as the inner-shell rich club (density: 0.97 (0.92–1.03)), and the outer-shell rich club (density: 1.01 (0.99–1.04)) of the fly connectome. The visual-auditory and the inner-shell rich clubs are largely localized within modules (Fig. 1b, Supplementary Table 1), and so it is unsurprising that constrained intra-module weights enforced these rich-club densities to empirical levels. In contrast, the default-mode and the outer-shell rich clubs are not localized to specific modules and are therefore not subject to the same mechanism. Most interestingly, the default-mode rich club is comprised of nodes which, by virtue of their highly distributed connections, could not be assigned to any modules and could not therefore be directly affected by constrained intra-module weight (Fig. 1b, Supplementary Table 1a). The accurate reproduction of this rich-club density is likely to arise from indirect effects of combined module and hub constraints. The many strong connections of hub nodes cannot be equally distributed between modules (to maintain constrained intra-module weights) and must therefore be preferentially placed between other hubs, enforcing the connectivity within this rich club to empirical levels.

### Robustness of results to differences in sampling

We evaluated the robustness of our results to differences in sampling, using an alternative sampling method. This alternative method supports weighted node and module constraints, but not wiring-cost constraints, nor simultaneous binary and weighted constraints; in addition, the method does not preserve empirical connection weights (Table 1). This method guarantees that imposed constraints are satisfied on average in the full network ensemble: Supplementary Fig. 4a–h shows decreases in constraint error associated with increases in sampled network ensemble size. We noted that 1,000-network ensembles had similar constraint errors to our primary sampling method (cf. Supplementary Fig. 1), and for ease of comparison, focused on averages of network measures (that is, on consensus module partitions and average rich-club densities) computed on these 1,000-network ensembles (for completeness, Fig. 5 also shows results computed on single networks). Note that averages of measures computed over 1,000-network ensembles were very stable, and thus mostly had negligible variance.

Results for the basic-hallmark model were robust to differences in sampling methods. Figure 5a,b shows that the basic-hallmark model sampled with the alternative method, similarly reproduced the high-resolution (NMI mouse: 0.88 (0.88–0.88), fly: 0.79 (0.79–0.79)) and low-resolution (NMI mouse: 0.75 (0.75–0.79), fly: 0.66 (0.66–0.66)) connectome module partitions. Furthermore, a simpler model with constrained intra-module weight but not node strength reproduced the low-resolution partition less accurately in the mouse (NMI: 0.30 (0.30–0. 30), Supplementary Fig. 6a) but more accurately in the fly (NMI: 0.88 (0.88–0.88), Supplementary Fig. 6b); future analyses of more highly resolved connectome reconstructions should help to resolve this discrepancy.

Figure 5c,d shows that the basic-hallmark model sampled with the alternative method reproduced all rich-club densities in both connectomes. Moreover, rich-club densities in these models were even higher than in the connectomes; especially for the mouse default-mode rich club (density: 1.45 (1.45–1.46)), and for the fly inner-shell rich club (density: 1.25 (1.24–1.26)). We investigated this effect in more detail by considering a third sampling method, which had properties intermediate to the primary and alternative methods. On the one hand, this method sampled networks with hard constraints and thus automatically preserved connection weights (similar to the primary method); on the other hand, this method matched only weighted, but not binary, constraints (similar to the alternative method). Basic-hallmark models sampled with this method had rich-club densities closer to empirical levels (default-mode rich club: 1.24 (1.16–1.32), inner-shell rich club: 1.00 (0.95–1.04)), suggesting that the absence of additional binary and connection-weight constraints surprisingly raised these densities to higher-than-empirical levels.

## Discussion

Our results have three important implications for future connectome analyses.

First, these results suggest that realistic wiring-cost constraints are insufficient to reproduce the connectome module organization, but that module hierarchies are structural byproducts of modules and hubs. These results diminish the perceived importance of economical wiring and module hierarchies on connectome organization. Such importance was posited, at least for vertebrate brains, in a modern version of Ebbesson's parcellation hypothesis^58^,^59^, which described that wiring pressures drive the hierarchical subdivision of larger and less differentiated brain areas into smaller and more differentiated brain areas, through selective connection loss. In an important counterbalance to this view, Deacon's displacement hypothesis^59^,^60^ emphasized a greater role for connectional ‘invasion', the propensity for disproportionately expanded brain areas to form evolutionary new connections. Our findings attribute greater importance to such displacement processes, although more comprehensive comparative analyses are clearly needed^61^. Our findings additionally prompt a substantial reformulation of the cost-efficiency hypothesis. As we already noted, the biologically interpretability of such reformulation would benefit from increased emphasis on empirical and anatomically specific features, and reduced emphasis on artificial or phenomenological models, or on generic measures.

**Figure 2:**
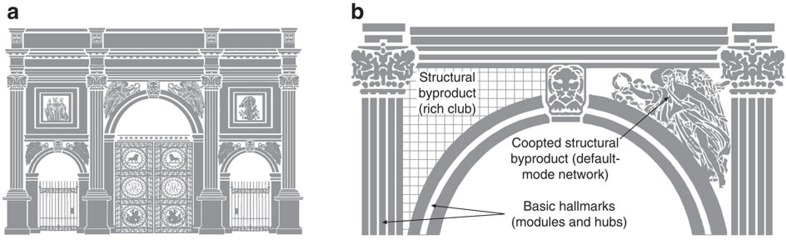
Architectural hallmarks, constraints and byproducts. (**a**) An elaborate architectural work, such as a triumphal arch, contains multiple features postulated to be primary or important for its overall functionality or design (such as curved arches and pillars). The simultaneous presence of such basic hallmarks necessarily constrain other parts of this architectural system. (**b**) Spandrels, the roughly triangular empty spaces induced by arch and pillar constraints (grid) are structural byproducts of such constraints, and an apt metaphor for equivalent byproducts in evolutionary biology. Structural byproducts may be nonfunctional, or may be evolutionarily coopted to perform new function (sculpture). The present study shows that the postulated basic hallmarks of modules and hubs induce the structural byproducts of hierarchies and rich clubs. The study posits that the default-mode network represents a structural byproduct coopted for higher cognition in humans.

**Figure 3 f3:**
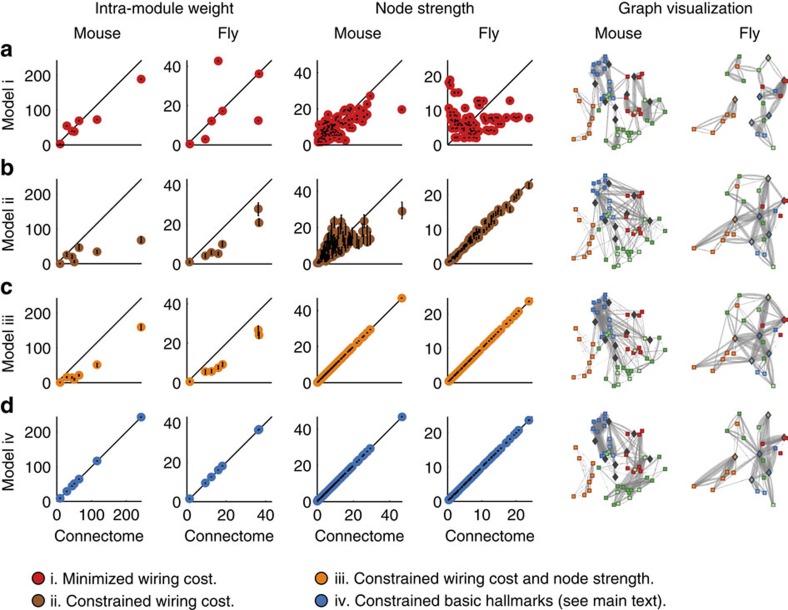
Wiring-cost constraints systematically underestimate intra-module weight in mouse and fly interareal connectome reconstructions. Left and middle: scatter plots of connectome versus model high-resolution intra-module weight and node strength for models with (**a**) minimized wiring-cost model, (**b**) constrained wiring-cost, (**c**) constrained wiring-cost and node strength and (**d**) constrained basic hallmarks, as described in the main text. Network models constrained by empirical levels of wiring-cost (**b**,**c**) had lower intra-module weight for all modules in both connectome reconstructions. All networks were sampled with the primary (hard-constraint) method. Bars show medians and interquartile ranges estimated from 100-sampled network models. All values for the fly connectome were divided by 1,000 for clarity of presentation. Right: graph visualizations of connections averaged over 100-network model samples, presented and coloured as in Fig. 1.

**Figure 4:**
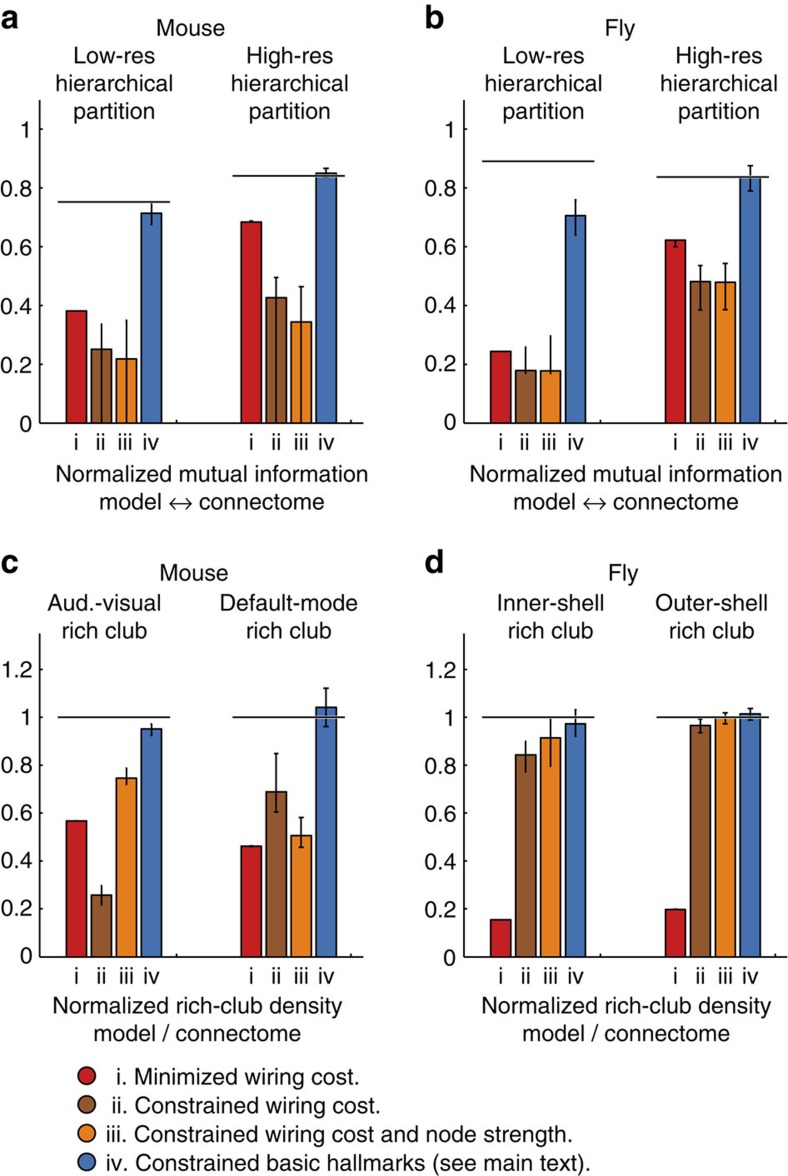
Module hierarchies and rich clubs are structural byproducts of basic connectome hallmarks. (**a**,**b**) NMI between model and connectome hierarchical module partitions. Horizontal lines indicate corresponding medians of benchmark models (cf. Supplementary Fig. 3a,b). (**c**,**d**) Normalized (model/connectome) rich-club densities. Horizontal lines indicate the rich-club density threshold of 1. The minimized-wiring-cost model approximated high-resolution module structure, but did not reproduce hierarchical or rich-club structure. The two wiring-cost-constrained models did not reproduce hierarchical or rich-club structure. In contrast, the basic-hallmark model reproduced hierarchical and rich-club structures in both connectome reconstructions. All networks were sampled with the primary (hard-constraint) method. Bars show the medians and interquartile ranges estimated from 100-network model samples.

**Figure 5:**
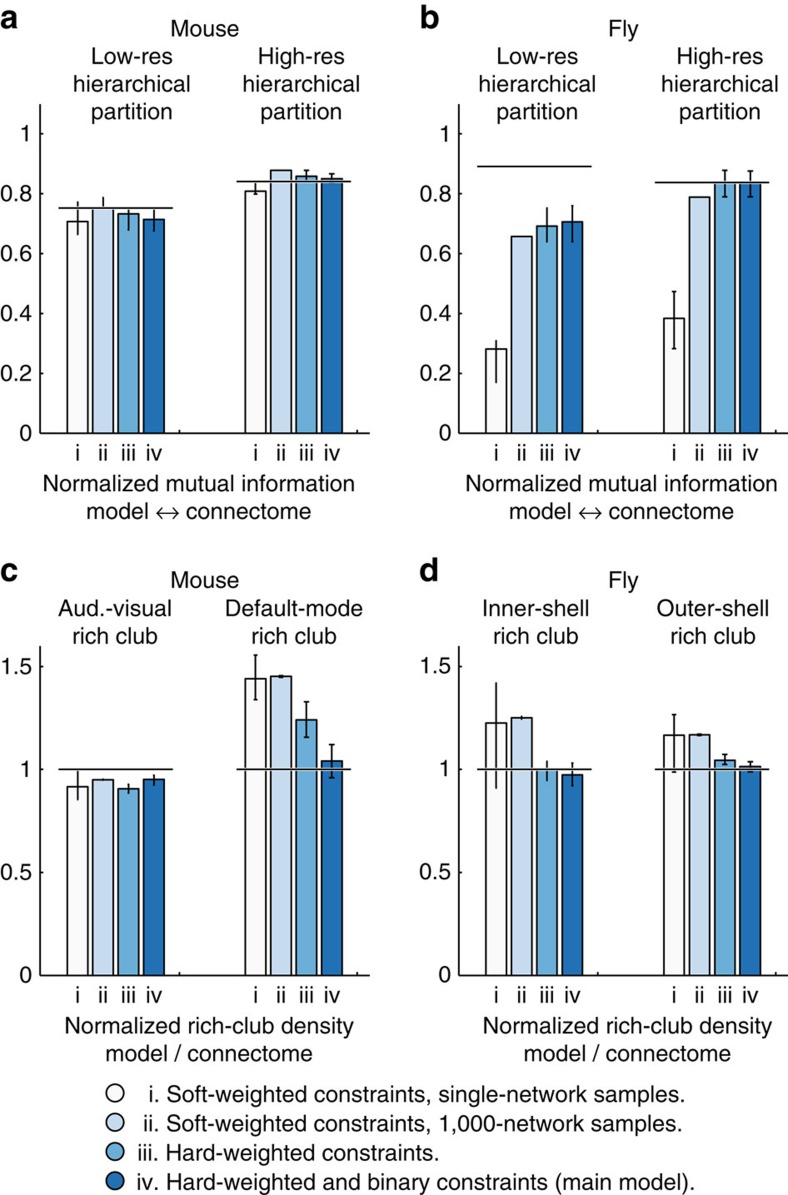
Results for the basic-hallmark model are robust to sampling methods. Basic-hallmark model sampled with the primary (hard-constraint) and alternative (soft-constraint) methods (**a**,**b**) NMI between model and connectome hierarchical module partitions. Horizontal lines indicate medians of benchmark models (see also Supplementary Fig. 3a,b). (**c**,**d**) Normalized (model/connectome) rich-club densities. Horizontal lines indicate the rich-club density threshold of one. Averages of network measures from 1,000-network ensembles sampled with the alternative method (model ii) reproduced the hierarchical and rich-club structures in both connectome reconstructions. Moreover, rich-club densities of networks sampled with this method were even higher than rich-club densities of the connectomes. Bars show the medians and interquartile ranges estimated from 100-network model samples (for the primary method) or 100-network model ensembles (for the alternative method).

Second, our results suggest that rich clubs are structural byproducts of modules and hubs. These results may help to reconcile the conflicting association between the default-mode network and human cognition on one hand, and the presence of the default-mode network in mice and rats on the other hand. For instance, Braga and Leech^62^ hypothesized that the default-mode network may have originally evolved for a more basic homeostatic function, and was subsequently coopted for higher cognition in humans^63^. Our results simplify this hypothesis by removing the need to postulate such an adaptive function (Fig. 2b). Buckner and Krienen^64^ hypothesized that the disproportionate cortical expansion in humans resulted in the formation of densely interconnected cortical association areas freed or ‘untethered' from developmental (for example, axonal guidance) constraints. Our results add to this hypothesis by noting that the putative propensity for association areas to form many connections, through connectional displacement or similar processes (‘hub constraints'), together with the evolutionarily conserved architecture of sensory, motor and homeostatic systems (‘module constraints') necessarily leads to disproportionately dense connectivity between association areas (‘rich-club byproducts'). Our observations on the mouse suggest that such effects may represent a more general feature of the mammalian brain, potentially accentuated in humans through disproportionate expansion of the late-developing association cortex^65^.

Third, our results imply that the currently standard reports of simultaneously present modules, hubs, hierarchies and rich clubs engage in circular analysis or double dipping. Constraint network models emphasize an integrative approach to connectome analysis and provide an empirically grounded framework for avoiding similar pitfalls in future studies. We used these models to reduce the stipulated principles of connectome organization from the set of modules, hubs, hierarchies, rich clubs, and wiring-cost/efficiency trade-offs into a simpler and empirically grounded set of modules and hubs. An important benefit of our approach is the need to directly formulate the often tacitly made assumptions of basic connectome hallmarks. This direct formulation in turn emphasizes the need to test the biological and evolutionary basis for these assumptions in future studies. For example, our assumption for the importance of hubs, while presently ubiquitous and uncontroversial^19^, conflicts with knowledge that putative hubs in the mammalian diencephalon or in the insect central complex, contain diverse cytoarchitecturally or connectionally distinct subunits^66^,^67^. A subdivision of these areas in future more highly resolved connectome reconstructions may substantially alter the evidence for the existence or importance of interareal hubs. However, and in spite of any such future insights, pervasive interdependences between connectome features are likely to persist, and to require the types of analyses we emphasized in the present work.

## Methods

### Mouse interareal connectome data set

We studied the mouse interareal connectome reconstruction described in Rubinov *et al*.^8^ and based on data from 461 anterograde recombinant adeno-associated virus tracer injections in adult male wild-type C57BL/6J mice, registered into a common template brain and made available by the Allen Institute for Brain Science at http://connectivity.brain-map.org/ (ref. 4). The whole brain was subdivided into 56 bilaterally symmetric areas (a total of 112 areas), using a developmental ontology for extra-cortical areas from http://developingmouse.brain-map.org/ (refs 68, 69) and a topographic ontology for cortical areas from http://mouse.brain-map.org/ (ref. 70). See Supplementary Table 1a for all region names and Puelles *et al*.^68^ for comparison of topographic and developmental ontologies. Directed interareal projection weights were quantified as projection densities normalized by areal volumes; 6,542 connections were detected (53% connection density) and connection weights scaled over several orders of magnitude (Supplementary Fig. 4i). Interareal distances of white-matter pathways were quantified with deterministic tracer tractography, and tract bandwidth, a measure of pathway capacity, was quantified as the normalized estimated pathway cross-section^8^. All injections were right sided, and whole-brain connectome reconstruction assumed hemispheric symmetry.

### *Drosophila* interareal connectome data set

We studied the *Drosophila* interareal connectome reconstruction described in Shih *et al*.^10^, and based on data for 12,995 GFP-labelled projection neurons in adult female *Drosophila*, registered into a common template brain and made available as part of the FlyCircuit database at http://www.flycircuit.tw/ (ref. 9). The whole brain was subdivided into 23 bilaterally symmetric areas and 3 unpaired areas of the central complex (a total of 49 areas), using detection of local processing units, anatomically contiguous areas with self-contained local-neuron populations^9^. See Supplementary Table 1b for all region names and Ito *et al*.^71^ for comparisons with other anatomical nomenclatures. Directed interareal connection weights were quantified as the mean number of dendritic and axonal terminals for each neuron summed over all neurons for each area; 1,950 connections were detected (83% connection density), and connection weights scaled over several orders of magnitude (Supplementary Fig. 4j). For the present study, we additionally quantified interareal distances of white-matter pathways with deterministic tracer tractography along masks of white-matter pathways from the FlyCircuit database, which we affinely registered to the JFRC2 adult *Drosophila* brain template^72^ made available by the Virtual Fly Brain resource at http://www.virtualflybrain.org/ (ref. 73). In the absence of pathway cross-section information, we equated tract bandwidth with pathway connection weight.

### Constraint network models

We represent each network by its directed weights matrix **W**={*w*_*ij*_} and denote the special case of the connectome itself by 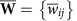. We seek to generate ensembles of random networks which satisfy *k* types of constrains imposed by node, module, wiring-cost or other network features. We represent each network feature by 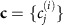, where *i* is the index on the type on constraint (for example, node strength) and *j* is the index of each constrained element (for example, the strength of node *j*). We denote the special case of connectome features we seek to constrain by 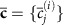.

The node strength is defined as


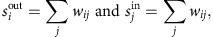


where the sum is over all nodes in the network.

The node wiring cost is defined as


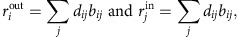


where *d*_*ij*_ is the length and *b*_*ij*_ is the bandwidth, of directed white-matter pathway from *i* to *j*.

The total weight between modules **u** and **v** is defined as


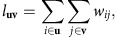


where the sums are over nodes in both modules; *l*_**uu**_ is the intra-module weight of **u**.

We constrained node strengths and wiring costs for all nodes, and we constrained intra- and inter-module weights for all modules, whenever these constraints were imposed. In addition, our primary sampling method preserved weighted and binary variants of all constraints, while our alternative sampling method preserved only weighted variants of all constraints (Table 1). We computed binary variants of all constraints by substituting all weights *w*_*ij*_ (and independently all bandwidths *b*_*ij*_) with adjacencies *a*_*ij*_ representing the presence (*a*_*ij*_=1) or absence (*a*_*ij*_=0) of a connection from node *i* to node *j*.

### Primary network sampling method

This sampling method is based on constrained randomization of empirical networks and aims to uniformly sample networks with hard constraints, such that each individual sampled network in the ensemble satisfies the constraints exactly^54^. Each network in the ensemble is sampled by randomizing the connectome, while numerically minimizing the constraint error *E*,


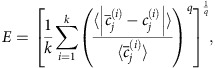


where 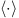 is the arithmetic mean over all constraints of type *i* (for example, node strength), and *q* defines the type of error. *E* is essentially a generalized mean, 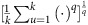, of the mean absolute constraint error, 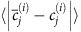, normalized by individual constraint magnitude, 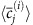. In this study, we used *q*=2, and thus minimized the mean squared error; the choice of *q* does not affect the global minimum of *E*, and exploratory analyses using the mean absolute error (*q*=1), or the maximum absolute error (*q*→∞), did not our change results. We also note that the connectome satisfies *E*=0 for all combinations of *q* and constraints 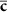; this underscores the empirical grounding of constraint network models.

We minimized the constraint error using simulated annealing, a popular metaheuristic search algorithm motivated by the analogy of cooling a physical system. Global minimization with simulated annealing is implemented by iteratively swapping random pairs of connections with probability 1 if such swaps lower the error (Δ*E*<0), or with probability exp(−Δ*E/T*) otherwise. At the start of each search, the parameter *T* (‘temperature' in the physical analogy) is very high, such that effectively all swaps are accepted, and the system is randomized without constraints. As the search proceeds, *T* is slowly reduced (‘the system is cooled' in the physical analogy), such that swaps which raise the error are less likely to become accepted. Towards the end of the search, *T* is very low, such that effectively only swaps which reduce the error are accepted.

The simulated annealing search satisfies the conditions of ergodicity (that is, it can reach all possible network states), and reversibility (that is, all swaps are potentially reversible), and consequently, with a sufficiently slow lowering of *T* (‘cooling schedule' in the physical analogy), it is guaranteed to escape local minima, and to reach all global minima with equal probability—in other words to uniformly sample all networks exactly matching the specified constraints^74^. However, in practice it is not possible to say precisely what constitutes a sufficiently slow cooling schedule for each system; moreover, it is likely that sufficiently slow cooling schedules are computationally impractical. Here we set a more realistic goal of sampling networks with a small error, *E*<0.005, comparable to 0.5% deviation from the global minimum *E*=0 (Supplementary Fig. 1).

Our search starts by shuffling the empirical connectome, with preserved bilateral weight relationships or symmetry. We preserved bilateral weight relationships, since the bilateral symmetry assumption was inherent in the weight reconstruction of the mouse connectome (above), and since approximate bilateral symmetry was also observed in the *Drosophila* connectome (Spearman correlation of 0.82 between bilaterally symmetric connections).

We used a slow cooling schedule, initially setting *T*=1, which in practice allows network randomization without constraints. At every iteration, we randomly swapped two connection weights, and their bilaterally symmetric equivalents, and accepted this swap with 


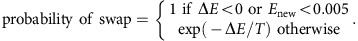


We ran the algorithm for 10^9^ iterations, and slowly reduced the temperature, as *T*_new_←0.999*T*, at every 10^4^ iterations.

### Alternative network sampling method

This sampling method is based on exact maximum-likelihood estimation of maximum-entropy/exponential random-graph models, and aims to unbiasedly sample networks with soft constraints, such that the constraints are exactly satisfied for the full network ensemble average, but not for each individual network^55^. For each connectome matrix 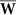 and set of constraints 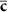, we sample constraint models **W** from a probability *P*(**W**). It can be shown^75^ that the only unbiased choice of probability P(**W**) in this case is the one which maximizes the entropy *S*,


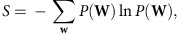


subject to the constraints


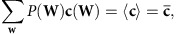


where **c**(**W**) is the value of **c** computed in network **W**, and 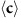 is the average of all **c**(**W**) in the network ensemble.

It is possible to derive the expression for the unbiased probability *P*(**W**), by introducing for each constraint 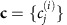 auxiliary variables, known as Lagrange multipliers, 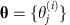, using these variables to write the entropy with constraints as a single expression, differentiating this expression with respect to *P*(**W**), and setting the derivative to 0. This gives


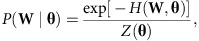


where the function 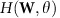, also known as the graph Hamiltonian, enforces the constraints


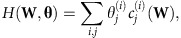


and the normalization constant *Z*(**θ**), also known as the partition function, is given by


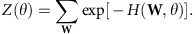


A binary version of these models has been extensively studied under the name of p* or exponential random-graph models^56^,^57^. Here, we make use of recent results which show that for first-order constraints (which include node-strength and module-weight, but not wiring-cost constraints), it is possible to derive exact expressions for the maximum-likelihood values of **θ**, solve these expressions numerically and thus estimate the maximum likelihood of *P*(**W**) (ref. 55). We summarize these results below and refer the reader to a detailed exposition of these results in ref. 55.

In the most general case of node-strength and module-weight constraints, with **u** and **v** denoting the modules of node *i* and *j* respectively, *H*(**W**, **θ**) may be factorized as:


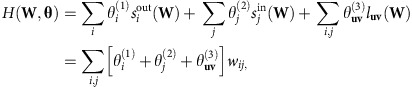


and the normalization constant *Z*(**θ**) may be written exactly as


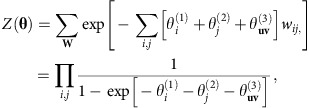


where we assumed integer weights without loss of generality (real-valued weights can be linearly mapped to integers with arbitrary precision). Furthermore, the probability *P*(**W**|**θ**) may be factorized as:


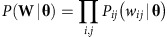


where 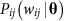, the conditional probability of each connection weight, is expressed as:


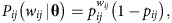


with


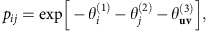


such that each individual (non-negative) integer weight *w*_*ij*_ is essentially sampled from the so-called geometric distribution 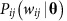 (ref. 76).

After introducing auxiliary variables, 
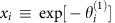
, 
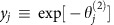
, and 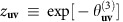, the expectation of each geometrically distributed weight 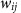 can be written analytically as:


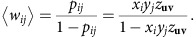


The constraints can now be directly expressed in terms of weight expectations,


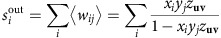



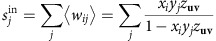



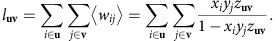


This coupled system of nonlinear equations is easily solved with modern numerical software (we used MATLAB's fsolve). This solution is equivalent to the maximum-likelihood estimate for **θ**, and thus finally gives us the unbiased probabilities of individual connection weights *P*_*ij*_(*w*_*ij*_).

### Cost-minimized lattice model

We compared constraint models with the widely studied wiring-cost-minimized generative model, constructed by assigning progressively stronger weights to shorter pathways. To robustly assign weights to connections with similar lengths, we perturbed the estimated distance of each pathway by uniform noise on the order of 1% median connection distance; in practice this had negligible effect on the final results.

### Assessment of module hierarchies

Similarly to the original analyses of these connectome reconstructions, we detected module hierarchies using a multistep metaheuristic optimization algorithm based on maximization of the modularity statistic^77^,


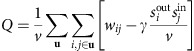


where *γ* is a module-resolution parameter (such that higher *γ* results in smaller modules) and *v* is a normalization constant (equivalent to the total network weight).

We detected characteristic module partitions for each *γ*=0.5, 0.55,…, 2.5 by performing consensus clustering^78^ on 1,000 local-maxima partitions^79^ obtained at each value of *γ* (ref. 80). We defined partitions as stable if they did not change for an interval of length ≥0.2 (for example, for an interval of 1.0≤*γ*≤1.2). These criteria ensured that the detected modules were robust to a range of module-resolution parameter values. In the mouse connectome we detected a two-level hierarchical organization, equivalent to our original analysis of these data (Fig. 1b, Supplementary Table 1a)^8^. In the fly connectome we also detected a two-level hierarchical organization as in the original analysis of these data (Fig. 1b, Supplementary Table 1b)^10^; however, in contrast to the original analysis, which simply assumed the presence of a stable low-resolution partition at *γ*=1, we detected a low-resolution partition at a slightly higher 1.15≤*γ*≤1.3. For consistency with the original analysis, we fixed *γ*=1 for the low-resolution partition of the fly connectome and network models, noting that the precise choice of *γ* for this partition did not change our results.

We compared the accuracy of module hierarchies in each network model, by applying the module detection algorithm to each model, and quantifying the similarity of stable partitions, closest in *γ* to the low-resolution and high-resolution partitions of each connectome. We quantified partition similarity with the NMI, a widely used information-theoretic measure of partition similarity defined as:


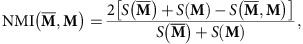


where 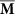 and **M** are connectome and model partitions, *S*(**M**) is the entropy and 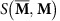 is the joint entropy. NMI is 1 for identical partitions, and 0 for maximally different partitions, or in the absence of stable partitions.

### Assessment of rich clubs

Rich clubs or cores are surprisingly highly connected groups of hub nodes^81^. Analyses demonstrated the presence of two rich clubs in each of the mouse and fly connectomes (Fig. 1, Supplementary Table 1)^8^,^10^. We assessed the existence of rich clubs in network models by computing, for each rich club **R**, the normalized rich-club density^82^,


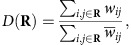


where *w*_*ij*_ and 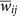 denote connection weights of the model and connectome respectively, and the sums are over all nodes in the rich club. This measure is equivalent to the inverse rich-club coefficient^81^. A network lacks a rich club when *D*(**R**)<1, and has a rich club when 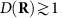.

### Code availability

Relevant code is available on request from the author, from the author's website (http://www.mikail-rubinov.net/), or from the Brain Connectivity Toolbox (http://www.brain-connectivity-toolbox.net/).

### Data availability

Mouse connectivity data was obtained and made available by the Allen Institute for Brain Science, USA (http://www.brain-map.org/). *Drosophila* connectivity data was obtained and made available by the National Center for High-performance Computing and National Tsing Hua University, Taiwan (http://www.flycircuit.tw/). Relevant data are available on request from the author.

## Additional information

**How to cite this article:** Rubinov, M. Constraints and spandrels of interareal connectomes. *Nat. Commun.* **7,** 13812 doi: 10.1038/ncomms13812 (2016).

**Publisher's note**: Springer Nature remains neutral with regard to jurisdictional claims in published maps and institutional affiliations.

## Supplementary Material

Supplementary Information Supplementary Figures 1-6 and Supplementary Table 1

## Figures and Tables

**Table 1:** Comparison of network sampling methods.

	**Primary method: constrained randomization of empirical networks**	**Alternative method: exact maximum-likelihood estimation of maximum-entropy/exponential random-graph models**
Type of sampling	Uniform sampling of networks with hard constraints: the constraints are satisfied with high accuracy for each individual sampled network.	Unbiased sampling of networks with soft constraints: the constraints are satisfied on average for the network ensemble, but not, in general, for each individual network.
Method of sampling	Specification of constraint-error function, and sampling of individual networks via numerical minimization (with simulated annealing) of this function.	Maximum-likelihood estimation of network probability distribution by numerical solution of systems of nonlinear equations, and sampling of individual networks directly from this distribution.
Type of studied constraints	Weighted and binary node-strength, module-weight, and wiring-cost constraints. In addition, all empirical connection weights are automatically preserved.	Weighted node-strength and module-weight constraints. Empirical connection weights are not preserved.
Accuracy	A small normalized constraint error (<0.005), similar for all network models.	Constraint errors are guaranteed to vanish in the limit of the full network ensemble. The studied 1,000-network ensembles had constraint errors similar in magnitude to the primary method.
Disadvantages	Uniform sampling is possible but not formally guaranteed.	Sampled distributions may not be representative of target distributions.
