# Supplementary material for: Constraints and spandrels of interareal connectomes
Source: Nat Commun. 2016 Dec 7;7:13812. doi: 10.1038/ncomms13812 (PMC5151054; doi:10.1038/ncomms13812)
Supplement: Supplementary Information — Supplementary Figures 1-6 and Supplementary Table 1 [file ncomms13812-s1.pdf]

# Supplementary Figure 1

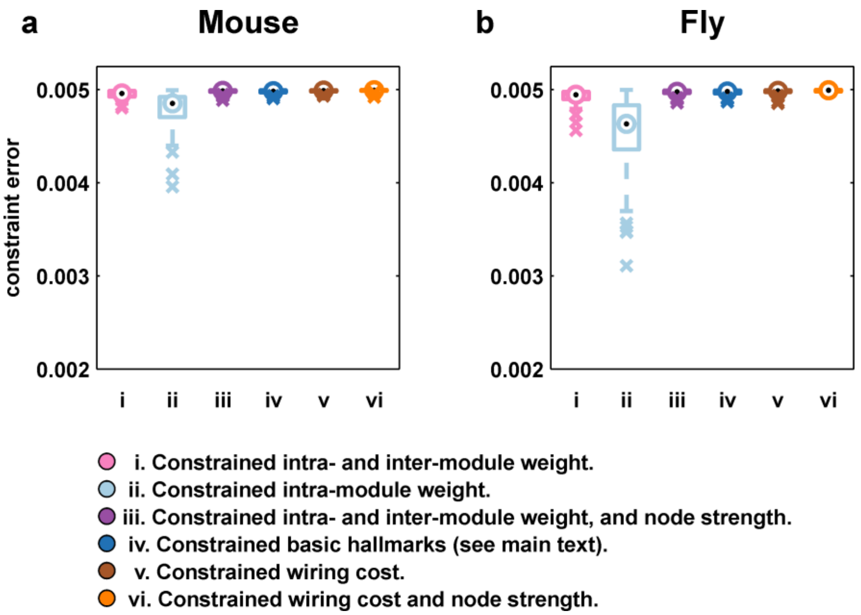


**Constraint errors of network models sampled with the primary (hard constraint) method.**

Box plots show normalized constraint errors for all sampled network models for the (a) mouse and (b) fly connectomes. Constraint errors were small and similar for all studied network models.

# Supplementary Figure 2

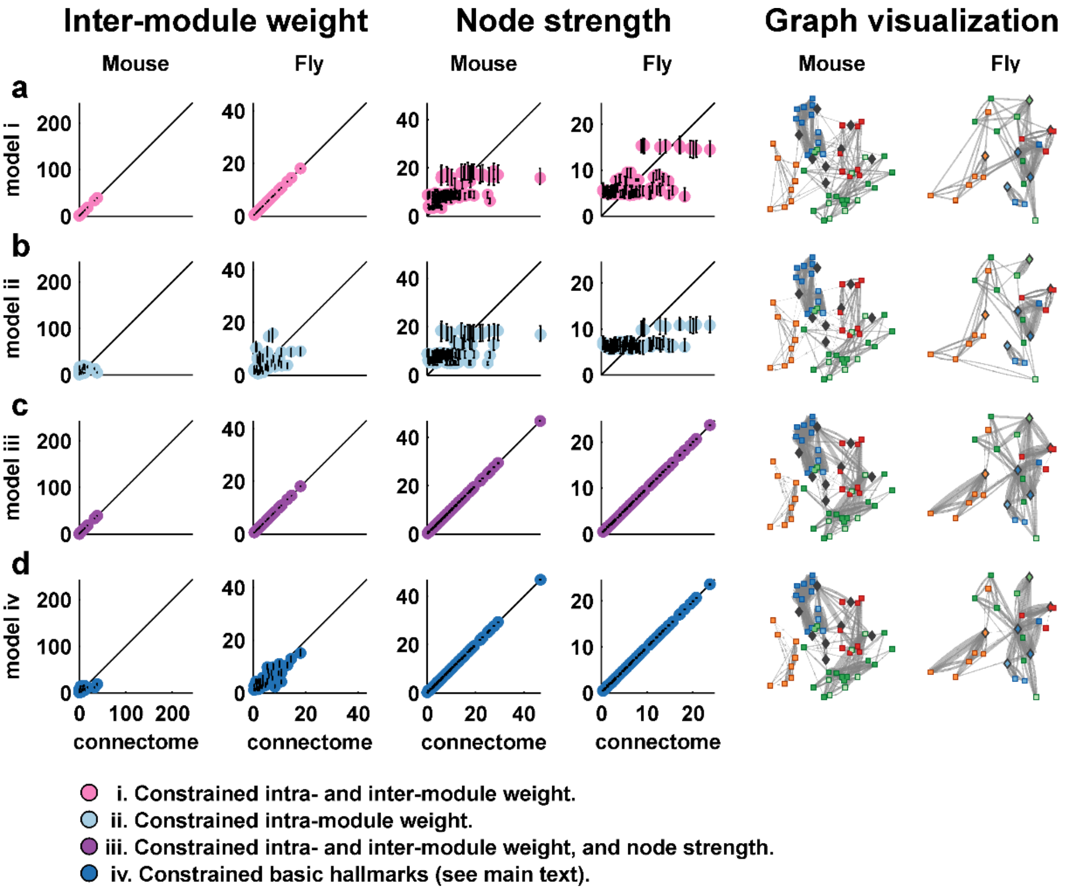


**Properties of basic-hallmark and related network models, sampled with the primary (hard constraint) method.** Left and middle: scatter plots of connectome vs model high-resolution *inter*-module weight and node strength for models with (a) constrained intra- and inter-module weight, (b) constrained intra-module weight, (c) constrained intra- and inter-module weight and node strength (the benchmark model used in the main text), and (d) constrained basic hallmarks, as described in the main text. Bars show the medians and interquartile ranges estimated from 100-network model samples. All values for the fly connectome were divided by 1000 for clarity of presentation. Right: graph visualizations of connections averaged over 100-network model samples, presented and colored as in Fig. 1.

## Supplementary Figure 3

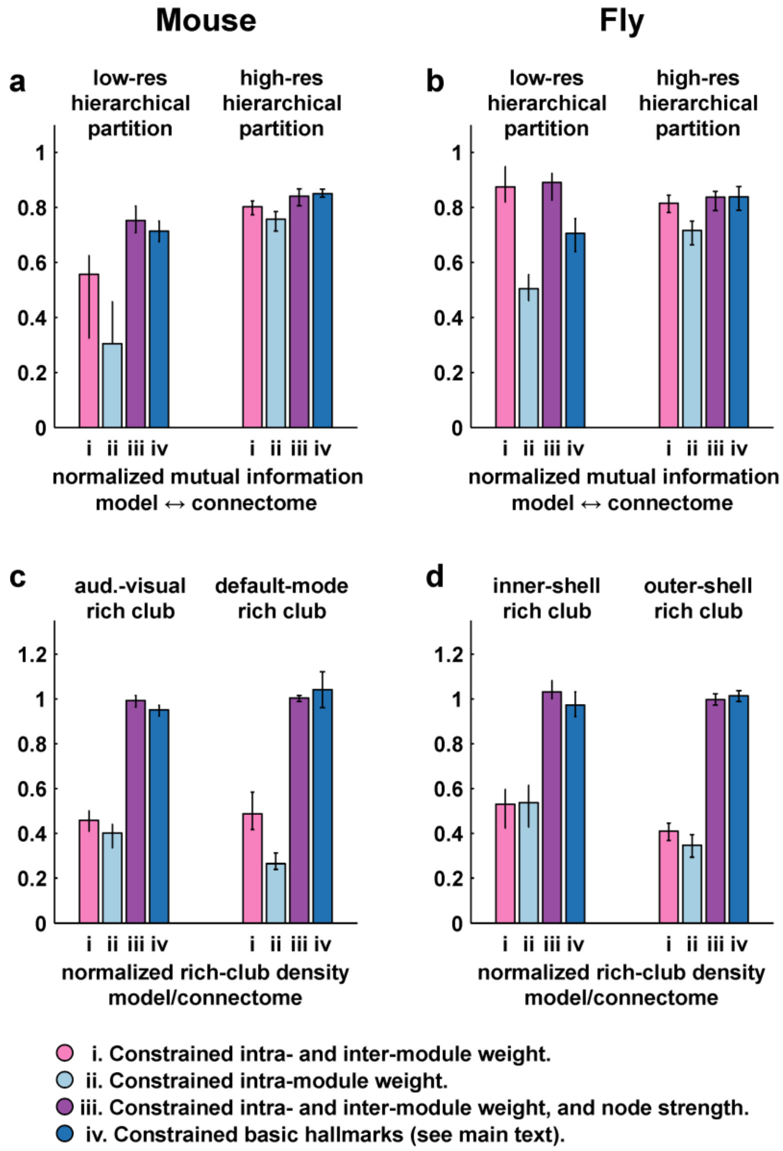


**Structural byproducts of basic connectome hallmarks and related models sampled with the primary (hard constraint) method.** (a-b) Normalized mutual information between model and connectome hierarchical partitions, and (c-d) Normalized (model/connectome) rich-club densities for the four network models of Supplementary Fig. 2. Model (iii) is the benchmark model used in the main text . Bars show the medians and interquartile ranges estimated from 100-network model samples.

# Supplementary Figure 4

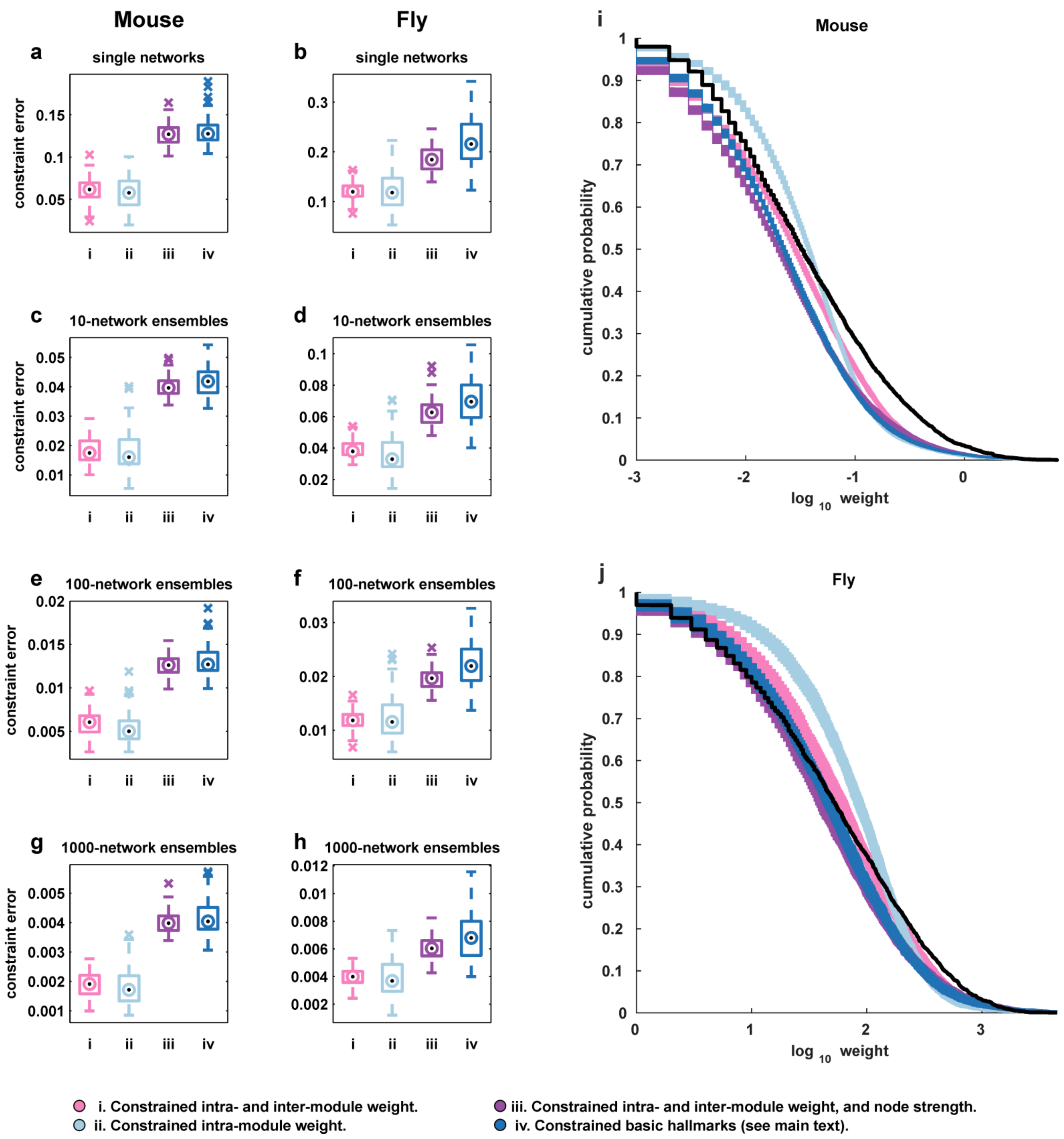

# Supplementary Figure 4 (continued)

**Constraint errors and weight distributions of network model ensembles sampled with the alternative (soft-constraint) method.** Box plots of normalized constraint errors for the (a, c, e, g) mouse and (b, d, f, h) fly connectomes. Note that 1000-network ensembles have constraint errors similar to those of individual network models sampled with the primary (hard-constraint) method (cf. Supplementary Fig. 1). (i-j) cumulative connection weight distributions for the connectome (black) and for the 1000-network ensembles sampled with the alternative (soft constraint) method (note the semilogarithmic scale).

## Supplementary Figure 5

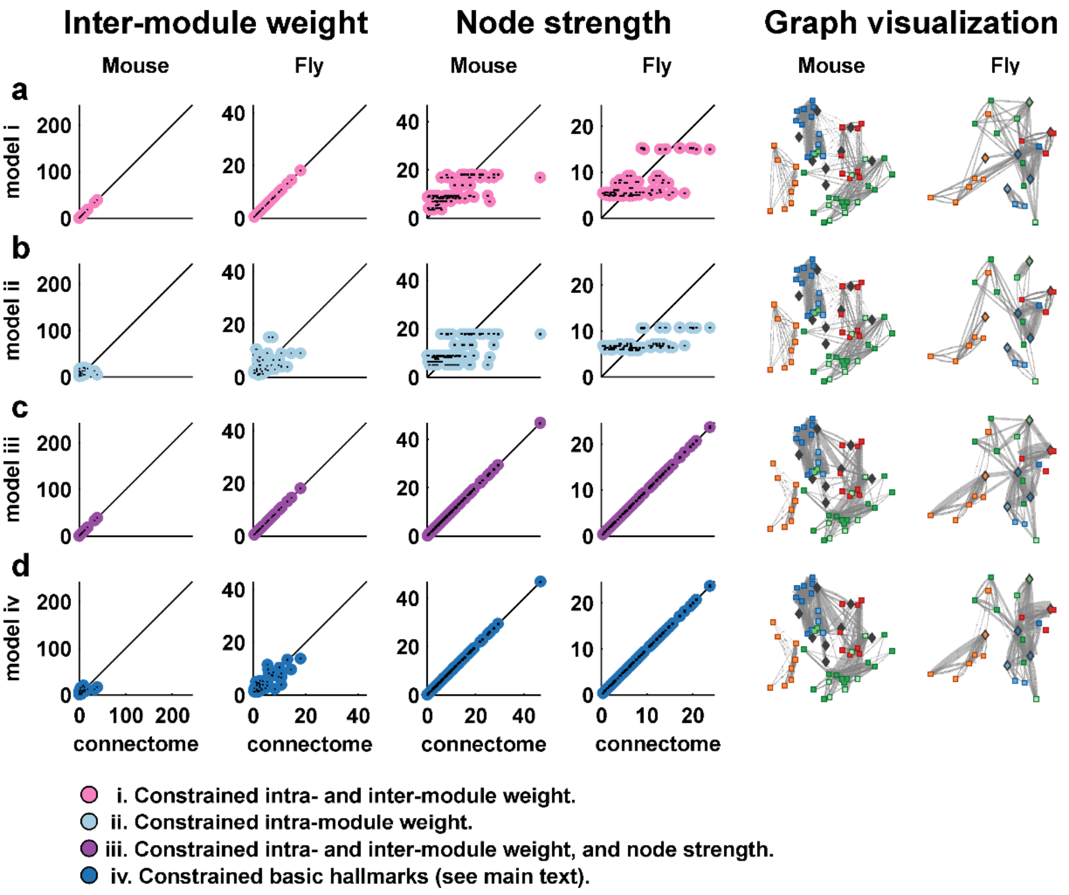


**Node and module properties of basic-hallmark and related network model ensembles, sampled with the alternative (soft constraint) method (1000-network ensembles).** Left and middle: scatter plots of connectome vs model high-resolution *inter*-module weight and node strength for models with (a) constrained intra- and inter-module weight, (b) constrained intra-module weight, (c) constrained intra- and inter-module weight and node strength, and (d) constrained basic hallmarks, as described in the main text. Bars show the medians and interquartile ranges estimated from 100-network model ensembles. All values for the fly connectome were divided by 1000 for clarity of presentation. Right: graph visualizations of expected connection weights for each type of network model, presented and colored as in Fig. 1.

## Supplementary Figure 6

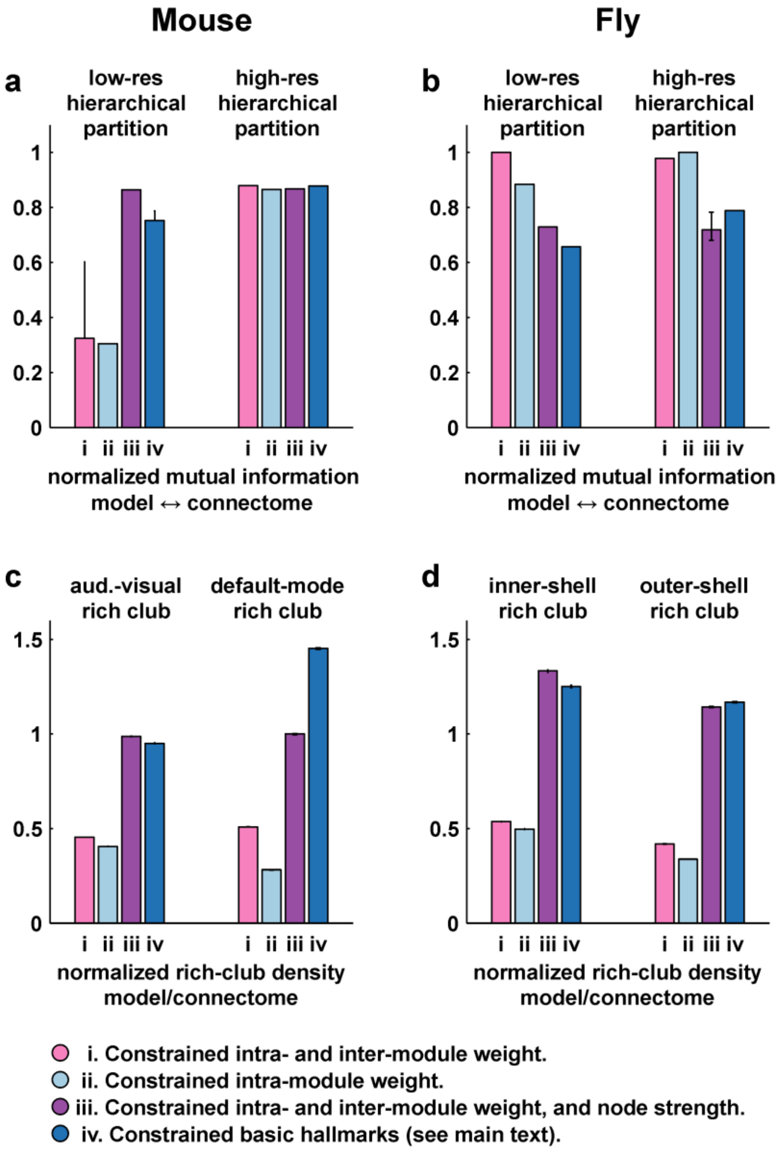


**Accuracy of basic connectome hallmarks and related models sampled with the alternative (soft constraint) method, (1000-network ensembles).** Normalized mutual information between model and connectome hierarchical partitions, and (c-d) Normalized (model/connectome) rich-club densities for the four network models of Supplementary Fig. 5. Bars show the medians and interquartile ranges estimated from 100-network model ensembles.

## Supplementary Table 1 (next two pages)

Definitions of (a) mouse and (b) fly connectome module hierarchies (nestings of high-resolution modules in low-resolution modules) and rich clubs (densely intra-connected groups of hub areas). Areas of the default-mode rich club in the mouse brain could not be assigned to specific modules. All areas, except ellipsoid body (EB), fan-shaped body (FB), and protocerebral bridge (PCB) in the central complex of the fly brain (asterisked), were bilaterally symmetric. The total number of areas was 112 for the mouse brain, and 49 for the fly brain.

| a. MOUSE | Area abbreviation | Area name | Low-resolution hierarchical partition | High-resolution hierarchical partition | Visual/ auditory rich club | Default-mode rich club |
|---|---|---|---|---|---|---|
| Brainstem/Cerebellar module | is | Isthmus | 1 | 1.1 | - | - |
| | CbV | cerebellar vermis | 1 | 1.1 | - | - |
| | r1 | rhombomere 1 | 1 | 1.1 | - | - |
| | CbH | cerebellar hemisphere | 1 | 1.1 | - | - |
| | r2 | rhombomere 2 | 1 | 1.1 | - | - |
| | PH | pontine hindbrain | 1 | 1.1 | - | - |
| | PMH | pontomedullary hindbrain | 1 | 1.1 | - | - |
| | MH | medullary hindbrain (medulla) | 1 | 1.1 | - | - |
| Visual/auditory module | RSP | Retrosplenial area | 2 | 2.1 | - | - |
| | VISp | Primary visual area | 2 | 2.1 | - | - |
| | VISpm | posteromedial visual area | 2 | 2.1 | - | - |
| | ECT | Ectorhinal area | 2 | 2.1 | - | - |
| | VISam | Anteromedial visual area | 2 | 2.1 | X | - |
| | VISal | Anterolateral visual area | 2 | 2.1 | X | - |
| | VISl | Lateral visual area | 2 | 2.1 | X | - |
| | VISpl | Posterolateral visual area | 2 | 2.1 | X | - |
| | TEa | Temporal association areas | 2 | 2.1 | X | - |
| | AUDp | Primary auditory area | 2 | 2.2 | - | - |
| | AUDd | Dorsal auditory area | 2 | 2.2 | - | - |
| | AUDv | Ventral auditory area | 2 | 2.2 | X | - |
| Somatosensory/ somatomotor module | AI | Agranular insular area | 3 | 3.1 | - | - |
| | SSp | Primary somatosensory area | 3 | 3.1 | - | - |
| | SSs | Supplemental somatosensory area | 3 | 3.1 | - | - |
| | VISC | Visceral area | 3 | 3.1 | - | - |
| | MOp | Primary motor area | 3 | 3.1 | - | - |
| | MOs | Secondary motor area | 3 | 3.1 | - | - |
| | GU | Gustatory areas | 3 | 3.1 | - | - |
| | Stri | intermediate stratum of Str | 3 | 3.1 | - | - |
| Olfactory/Hippocampal module | AOB | Accessory olfactory bulb | 4 | 4.1 | - | - |
| | AON | Anterior olfactory nucleus | 4 | 4.1 | - | - |
| | MOB | Main olfactory bulb | 4 | 4.1 | - | - |
| | TR | Postpiriform transition area | 4 | 4.1 | - | - |
| | TT | Taenia tecta | 4 | 4.1 | - | - |
| | COA | Cortical amygdalar area | 4 | 4.1 | - | - |
| | CTXsp | Cortical subplate | 4 | 4.1 | - | - |
| | RHP | Retrohippocampal region | 4 | 4.1 | - | - |
| | PIR | Piriform area | 4 | 4.1 | - | - |
| | Dg | diagonal domain | 4 | 4.1 | - | - |
| | Pal | pallidum (globus pallidus complex) | 4 | 4.1 | - | - |
| | ASPall | subpallial amygdala | 4 | 4.1 | - | - |
| | CA | Ammon's horn | 4 | 4.2 | - | - |
| | DG | Dentate gyrus | 4 | 4.2 | - | - |
| | ILA | Infralimbic area | 4 | 4.3 | - | - |
| | POTel | preoptic telencephalon | 4 | 4.3 | - | - |
| | THy | terminal hypothalamus (rostral hypothalamus) | 4 | 4.3 | - | - |
| | SeSPall | subpallial septum | 4 | 4.3 | - | - |
| | PaSe | paraseptal subpallium | 4 | 4.3 | - | - |
| | PHy | peduncular (caudal) hypothalamus | 4 | 4.3 | - | - |
| No module affiliation | ACA | Anterior cingulate area | - | - | - | X |
| | Strp | periventricular stratum of Str | - | - | - | X |
| | p3 | prosomere 3 | - | - | - | X |
| | p2 | prosomere 2 | - | - | - | X |
| | p1 | prosomere 1 | - | - | - | X |
| | m1 | mesomere 1 | - | - | - | X |
| | ORB | Orbital area | - | - | - | X |
| | PTLp | Posterior parietal association areas | - | - | X | X |

| b. FLY | Area abbreviation | Area name | Low-resolution hierarchical partition | High-resolution hierarchical partition | Inner-shell rich club | Outer-shell rich club |
|---|---|---|---|---|---|---|
| Visual module | LOB | Lobulla | 1 | 1.1 | - | - |
| | LOP | Lobulla Plate | 1 | 1.1 | - | - |
| | MED | Medulla | 1 | 1.1 | - | - |
| | OG | Optic Glomerulus | 1 | 1.1 | - | - |
| | OPTU | Optic Tubercle | 1 | 1.1 | - | - |
| | VLP-D | Ventrolateral Protocerebrum, Dorsal part | 1 | 1.1 | X | X |
| | VLP-V | Ventrolateral Protocerebrum, Ventral part | 1 | 1.1 | - | - |
| Auditory/ mechanosensory module | CCP | Caudalcentral Protocerebrum | 2 | 2.1 | - | - |
| | DMP | Dorsomedial Protocerebrum | 2 | 2.1 | X | X |
| | VMP | Ventromedial Protocerebrum | 2 | 2.1 | X | X |
| | AMMC | Antennal Mechanosensory and Motor Center | 2 | 2.2 | - | - |
| | CMP | Caudalmedial Protocerebrum | 2 | 2.2 | - | - |
| | CVLP | Caudal Ventrolateral Protocerebrum | 2 | 2.2 | - | X |
| Pre-motor module | EB* | Ellipsoid Body | 3 | 3.1 | - | - |
| | FB* | Fanshaped Body | 3 | 3.1 | - | X |
| | IDFP | Inferior Dorsofrontal Protocerebrum | 3 | 3.1 | - | - |
| | NOD | Noduli | 3 | 3.1 | - | - |
| | PCB* | Protocerebral Bridge | 3 | 3.1 | - | - |
| Olfactory module | AL | Antennal Lobe | 4 | 4.1 | - | - |
| | DLP | Dorsolateral Protocerebrum | 4 | 4.1 | - | - |
| | LH | Lateral Horn | 4 | 4.1 | - | - |
| | PAN | Proximal Antennal Protocerebrum | 4 | 4.1 | - | - |
| | SPP | Superpenduncular Protocerebrum | 4 | 4.1 | - | - |
| | MB | Mushroom Body | 4 | 4.2 | - | - |
| | SDFP | Superior Dorsofrontal Protocerebrum | 4 | 4.2 | - | X |
| | SOG | Subesophageal Ganglion | 4 | 4.3 | - | - |
